# Thymol tolerance in *Escherichia coli* induces morphological, metabolic and genetic changes

**DOI:** 10.1186/s12866-019-1663-8

**Published:** 2019-12-16

**Authors:** Fatemah Al-Kandari, Rabeah Al-Temaimi, Arnoud H. M. van Vliet, Martin J. Woodward

**Affiliations:** 1 Department of Food and Nutrition Science, School of Chemistry, University of Reading, Reading, RG6 6AP UK; 2 Department of Plant Protection, Public Authority Of Agriculture Affairs & Fish Resources, Al-Rabia, Kuwait; 3 Human Genetics Unit, Department of Pathology, Faculty of Medicine, Kuwait University, Jabriya, Kuwait; 4 Department of Pathology and Infectious Diseases, School of Veterinary Medicine, Faculty of Health and Medical Sciences, University of Surrey, Guildford, GU2 7AL UK

**Keywords:** *Escherichia coli*, Thymol, Resistance, Acriflavine resistance regulator, Efflux pump

## Abstract

**Background:**

Thymol is a phenolic compound used for its wide spectrum antimicrobial activity. There is a limited understanding of the antimicrobial mechanisms underlying thymol activity. To investigate this, *E. coli* strain JM109 was exposed to thymol at sub-lethal concentrations and after 16 rounds of exposure, isolates with a 2-fold increased minimal inhibitory concentration (MIC) were recovered (JM109-Thy^r^). The phenotype was stable after multiple sub-cultures without thymol.

**Results:**

Cell morphology studies by scanning electron microscopy (SEM) suggest that thymol renders bacterial cell membranes permeable and disrupts cellular integrity. ^1^H Nuclear magnetic resonance (NMR) data showed an increase in lactate and the lactic acid family amino acids in the wild type and JM109-Thy^r^ in the presence of thymol, indicating a shift from aerobic respiration to fermentation. Sequencing of JM109-Thy^r^ defined multiple mutations including a stop mutation in the *acrR* gene resulting in a truncation of the repressor of the AcrAB efflux pump. AcrAB is a multiprotein complex traversing the cytoplasmic and outer membrane, and is involved in antibiotic clearance.

**Conclusions:**

Our data suggests that thymol tolerance in *E. coli* induces morphological, metabolic and genetic changes to adapt to thymol antimicrobial activity.

## Background

The antimicrobial activity of many essential oils (EOs) such as thymol and carvacrol has been widely demonstrated [1, 2] and is assigned to a number of small terpenoid and phenolic compounds [3]. Thymol (C_10_H_14_O) is a monoterpenoid phenol extracted from thyme (*Thymus vulgaris*) as well as other plants. Thymol has been shown to have a wide range of potential applications in pharmaceuticals and therapeutics due to its effective anti-inflammatory, anti-oxidant, and anti-hyperlipidemic properties [4]. In the agriculture and food industry thymol has shown potential insecticidal and antimicrobial properties [5, 6]. Despite a large body of literature supporting the potential antimicrobial control of EOs and their minimal negative effects on human health, there are still relatively few applications in real foods due to lack of systematic studies of the single constituents of EOs and their effects either in model or real systems. However, there is some information on the mechanisms of action of these bioactive molecules, for example against food-borne microorganisms [7, 8]. Indeed, a deeper understanding of the microbial targets of EOs and their components as well as related microbial defence systems involved may permit a greater use of these antimicrobials in foods and food production. Recent studies have reported proteomic, genomic and metabolomic approaches to study pathogen cellular processes and their response to antibiotic stimuli [9, 10]. These approaches could identify the mode of action of thymol against *E. coli*.

Antibiotic resistance is a major cause for global burden on health, costs, and gross domestic products [11, 12]. *E. coli* antimicrobial resistance has been shown to be most prevalent in the agricultural industry imposing substantial threats to health and production [13, 14]. Several studies have shown EOs, especially thymol, can efficiently inactivate pathogens [2, 7, 15–17] but only a few provide insight into the EO mechanism of action. Burt and Reinders showed morphological changes in *E. coli* O157 caused by thymol [15], whereas Yuan et al. showed that thymol tolerance induced an altered expression profile supportive of resistance to thymol, heat, and oxidative stress in *E. coli* 0157 [8]. Currently, there are many antibiotic resistance mechanisms reported stemming from genetic and proteomic investigations in a wide range of pathogens [18, 19]. However, EOs effects in susceptible pathogens relevant to the food industry has not been equally studied [20]. More specifically, comprehensive analysis of changes in *E. coli* treated with thymol has not been performed. Therefore, the primary purpose of this research was to investigate the mechanism of action of thymol in *E. coli*.

## Results

### Adaptation of *E. coli* to thymol

The minimal inhibitory concentration (MIC) of thymol for *E. coli* JM109 was established prior to exposure to sub-inhibitory concentrations of thymol and was 175 μg l^− 1^. JM109 was shown to be tolerant of up to 3.5% ethanol, and the residual concentration of ethanol in the LB based thymol medium was 1%. The MIC of JM109 thymol-tolerant derivative (JM109-Thy^r^) was determined to be 400 μg l^− 1^ after 16 passages in gradual increasing concentrations of thymol. Tolerance to thymol was shown to be stable as demonstrated by repeated MIC tests in seven repeated subculture in LB broth without thymol (the JM109-Thy^r^ clone was passed through every 24 h for 7 days). After testing for stability, JM109-Thy^r^ clone culture was plated onto an NA plate and isolated colonies were used for subsequent experiments to assess JM109-Thy^r^ mechanism of resistance to thymol.

### Growth rate for JM109-Thy^r^

**Fig. 1:**
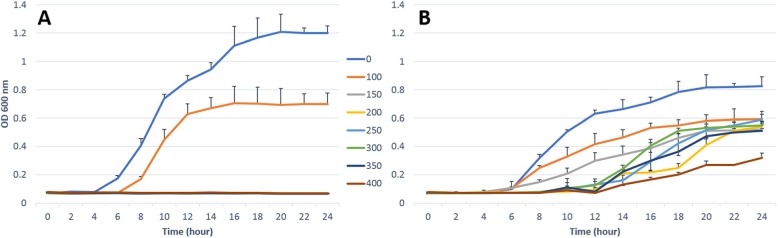
The effects of increasing concentrations of thymol on the growth of the wildtype JM109 *E. coli* (**A**), and JM109-Thy^r^ (**B**)

Figure 1A shows the significant growth differences between *E. coli* K12 laboratory strain JM109 and its JM109-Thy^r^ (*p* = 0.001). More specifically, the JM109-Thy^r^ when grown in LB without thymol showed a reduced growth rate and yield compared to control JM109 strain (Fig. 1B). In addition, the log and exponential phases were extended in high thymol concentrations to more than 20 h, and in most of the thymol concentrations tested it did not reach a stationary phase within the experimental time limit (24 h).

### Determination of *E. coli* morphology in the presence of thymol

**Fig. 2:**
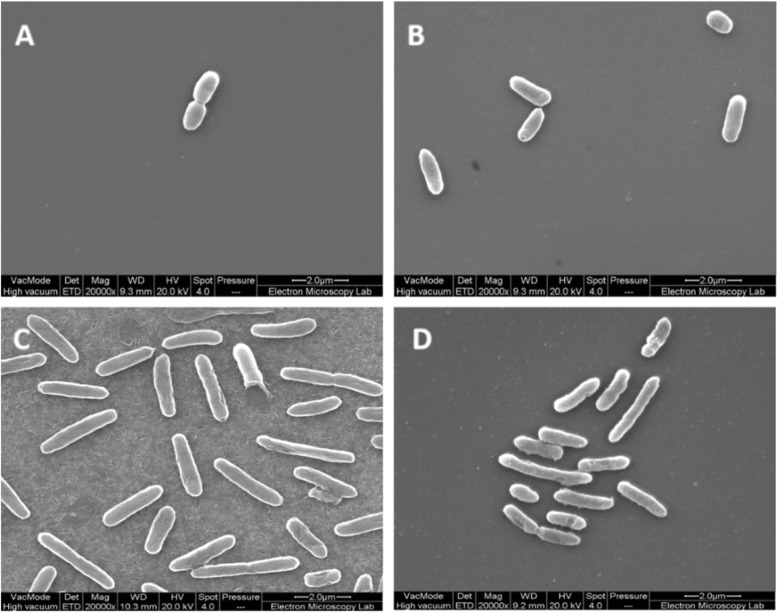
Scanning electron image of *E. coli* JM109 cells. (A) Thymol untreated wildtype JM109 cells; (B) JM109-Thy^r^ thymol untreated cells; (C) wildtype JM109 thymol treated cells; (D) JM109-Thy^r^ thymol treated cells

SEM analysis revealed JM109-Thy^r^ (Fig. 2B) displayed few morphological changes relative to wild-type (non-resistant) cells. Figure 2A shows JM109-Thy^r^ exhibited a slight corrugation of the cell surface and some cell body elongation. After exposure to sub-lethal concentrations of thymol at 50 μg l^− 1^, both tolerant and wild-type cells (Fig. 2C, D) showed morphological alterations in comparison to non-exposed cells (Fig. 2A, B). The wild-type JM109 had a uniform cylindrical shape and long cells with little evidence of septum formation. In the 23 whole cells analysed only two (8.7%) showed indications of septum formation. Besides these observations, the overall cell size of wild-type JM109 in the presence of thymol appeared larger than wild-type cells without thymol, and larger than JM109-Thy^r^ whether in the presence or absence of thymol. The average length of the wild-type strain grown in thymol was 1.57 μm whilst the average length of JM109-Thy^r^ strain was 1.3 μm (*p* = 0.01). In addition, JM109-Thy^r^ cells displayed more morphological changes after thymol challenge (Fig. 2D), the surface appeared to be ‘rough’ and showed irregularly shaped spots dotted along the cell body.

### Metabolic profile of JM109-Thy^r^

**Fig. 3:**
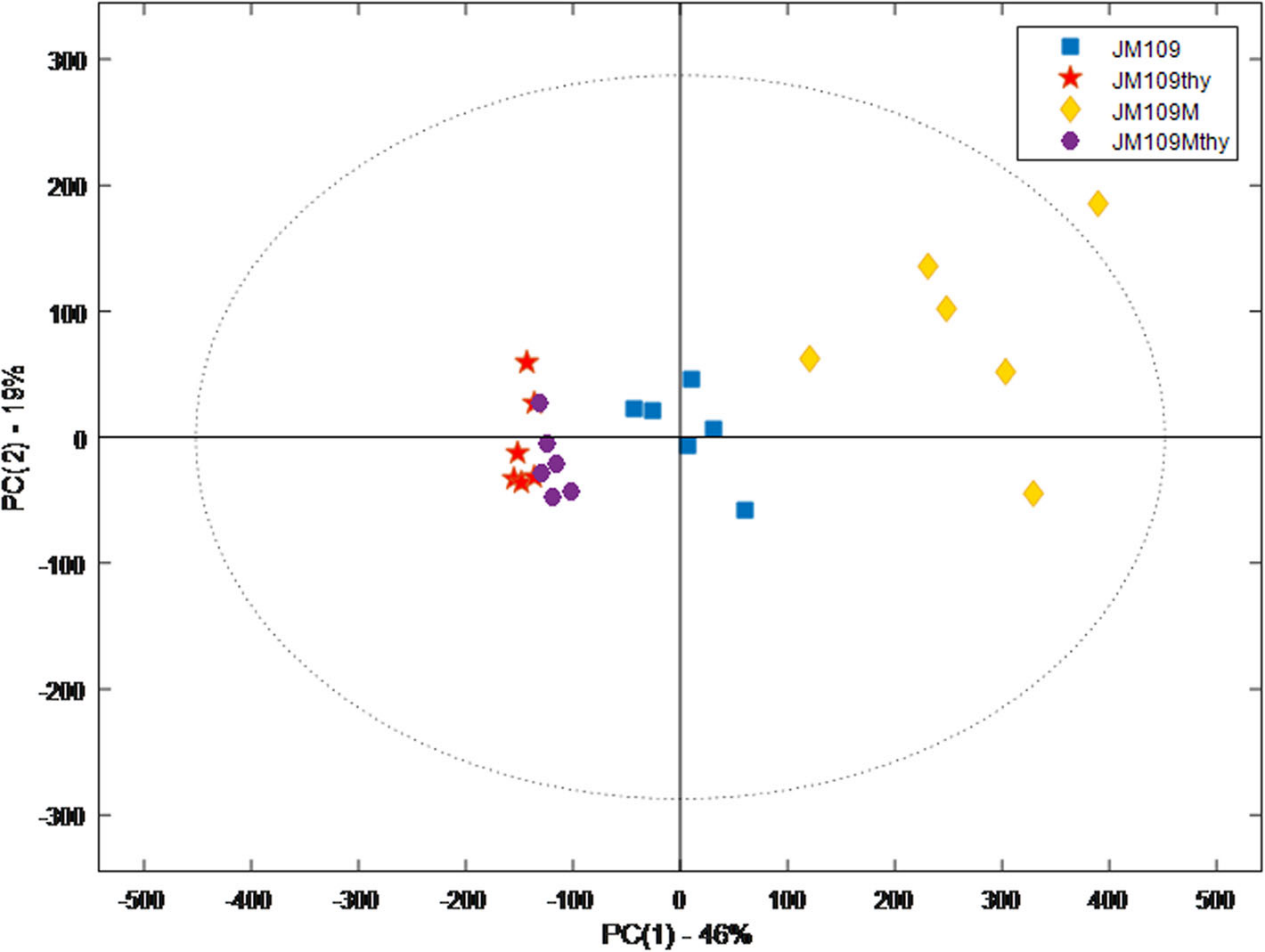
PCA-score plot illustrating the effect of different solvents on metabolic footprints derived from *E. coli* JM109 wildtype and JM109-Thy^r^ untreated and treated with a sub-lethal concentration of thymol (50μg l^− 1^). *N* = 6 for each sample (JM109thy: wildtype JM109 with thymol; JM109M: JM109 thymol tolerant derivative; JM109Mthy: JM109 tolerant derivative with thymol)

Orthogonal projection to latent structure (OPLS) is a powerful statistical modelling tool that provides insight into separations between experimental groups based on NMR high-dimensional spectral measurements. OPLS explained variation (R^2^Y) values around 0.8 were indicative of a good model, with Q2 values of ~ 0.5 indicative of good predictive ability. To analyse these complex data sets PCA analysis was performed (Fig. 3) which in this case summarises the original 65,536 variables detected. Thus, the direction and distance covered by the samples can be considered respective indicators of the differences between the metabolic profiles of each strain under the two test conditions, presence and absence of thymol. The metabolic profile of JM109 grown in M9 medium (n = six replicates) were tightly clustered indicating minimal sample to sample variation. However, the metabolic profile of the six replicates of JM109-Thy^r^ grown in M9 medium were more dispersed but discrete from JM109. It is clear that the metabolic profile of JM109-Thy^r^ strain was different from the wild-type, given the trajectory; suggests the presence of fewer small metabolites than wild-type. However, in the presence of thymol both wild-type and JM109-Thy^r^ were very comparable in their metabolic profile including very similar small metabolites.

**Fig. 4:**
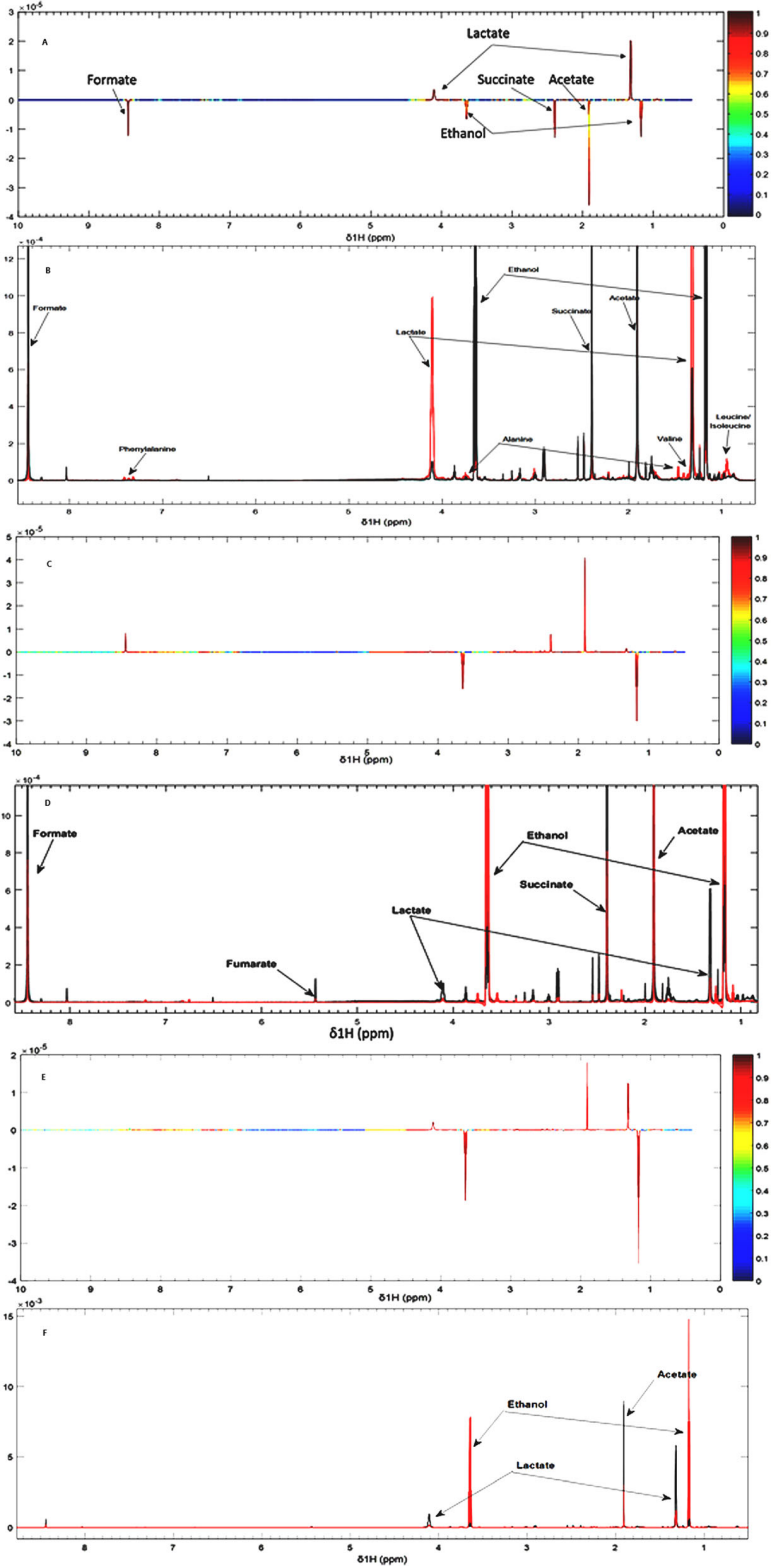
NMR spectra of JM109 wild-type and JM109-Thy^r^ strain grown with and without thymol. (A) S-line plot of wild-type JM109 (bottom) and JM109-Thy^r^ (top) grown without thymol, (B) partially assigned 700 MHz 1D spectra of wild-type (black) and JM109-Thy^r^ (red). (C) S-line plot of wild-type JM109 grown without thymol (top) and thymol treated (bottom), (D) partially assigned 700 MHz 1D-spectra of wild-type JM109 without thymol (black) and thymol treated (red). (E) S-line plot of JM109-Thy^r^ grown without thymol (top) and thymol treated (bottom), (F) partially assigned 700 MHz 1D-spectra of JM109-Thy^r^ grown without thymol (black) and thymol treated (red). Heat map indicates product concentration

PCA score plots also indicated differences in metabolic profiles of JM109 and JM109-Thy^r^. The comparison of wild-type and JM109-Thy^r^ grown in M9 without thymol (Fig. 4A**)** shows several peaks that correlate with energy metabolism end products (ethanol, formate, succinate and acetate) that were significantly higher in the wild type JM109 than JM109-Thy^r^. Succinate is the intermediary synthetic product of the tricarboxylic acid (TCA) cycle, whilst formate and acetate are the end products of the TCA cycle. These findings suggest JM109 wild-type respired aerobically. By contrast lactate was significantly higher in JM109-Thy^r^ than wild-type. Lactate is one of the main sugar fermentation products of *E. coli* that is produced by hydrogenation of pyruvate. Moreover, the aromatic amino acid phenylalanine and other amino acids, such as leucine, valine and alanine that belong to the pyruvate family of amino acids were produced more by JM109-Thy^r^ than by wild-type JM109 (Fig. 4B). Having identified metabolic differences between JM109 and JM109-Thy^r^ grown in M9 without thymol, we next examined the metabolic effects of thymol on both strains (Fig. 4C-F). A potential confounder of the data was the presence of 1% ethanol in both experiments as thymol was dissolved in ethanol and this molecule was therefore detected as a common feature in both strains. Thus, the production of ethanol by either strain would be masked by the excess already in the medium. In *E. coli* wild-type (Fig. 4C-D), the end products of glucose metabolism featured again but fumarate and lactate were also observed. In contrast, lactate was observed but at reduced concentrations along with acetate in JM109-Thy^r^ (Fig. 4E-F) suggesting slower growth in thymol possibly due to a shift from aerobic respiration to fermentation.

### JM109-Thy^r^ genetic changes

Having established a non-reverting, genetically stable JM109-Thy^r^ we sequenced its genome and compared it to the parental JM109 strain to identify mutations that may contribute to thymol tolerance. Results show that the parent and JM109-Thy^r^ strains both align to JM109 reference sequences. There were some major differences that could be ascribed to contig assembly and some regional inversions between the two strains. JM109-Thy^r^ strain harboured a JM109 backbone and was therefore a true derivative. Therefore, any mutations in specific genes are likely to be those that generate the phenotype observed. A mutation was identified in the *acrR* gene that encodes a repressor of AcrAB, which is a multidrug efflux pump. The mutation was a nonsense mutation converting an arginine residue at position 107 to a stop codon in the 215 amino acids long AcrR protein. The location of the mutation in *acrR* was a C to T transition at position 486,079 bases (gene size 485,761–486,408, locus tag = “b0464”) and abolishes a conserved amino acid residue in the C-terminal TetR domain. The other possible significant change was an Arginine to Cysteine amino acid change (R to C) at residue 118 in the ribonuclease G protein. The position of this mutation in the *rng* gene is − 3,397,444: *rng* (gene location 3,396,326–3,397,795 [reverse orientation], locus tag = “b3247”). Furthermore, an IS5 transposase gene had multiple silent point mutations, and the F-plasmid was missing in JM109-Thy^r^.

## Discussion

Widespread antibiotic resistance in bacterial species has lead scientists to pursue alternative natural products possessing antibacterial properties such as EOs. Thymol has been studied for its antimicrobial potential but many aspects of its mechanism of action have not been fully elucidated. Here, we propose a possible mechanism of action based on results of metabolomic and genomic investigation of an *E. coli* JM109-Thy^r^ isolate. *E. coli* JM109-Thy^r^ exhibited acquired sustained a stable tolerance to thymol after exposure to increasing sub-inhibitory concentrations of thymol, suggesting that in *E. coli* thymol tolerance could be the result of genetic mutation(s). It was noted that JM109-Thy^r^ had extended lag and exponential phases and a delayed stationary phase without thymol indicating that JM109-Thy^r^ strain had a reduced growth rate even in the absence of thymol. This finding is similar to other reports of slow bacterial growth in presence of terpenes to launch cellular survival and homeostasis mechanisms to survive EO antimicrobial action and regain replicative potential [21, 22]. Exposure to thymol conferred modest morphological changes in the cell wall and membrane of wild-type JM109 based on SEM analysis, whereas JM109-Thy^r^ displayed few morphological changes relative to wild-type cells. This suggests that thymol renders bacterial cell membranes permeable, which is similar to other studies that used EOs [15, 23–25]. Given these findings it may be postulated that thymol disrupts cell membrane structure and function including septum formation which is essential for cellular division and population growth. Also, since ion transport and ATP generation are located in the cell membrane these processes may also be disrupted. Collectively, these morphological alterations strongly suggest that gene regulatory processes may be coming into play perhaps to upregulate systems that detoxify thymol or prevent its entry, or/and increase fatty acid synthesis to repair cell membranes and so forth. This is an area for future research through transcriptomic approaches.

NMR results gave the first clues to the perturbation induced by thymol on *E. coli* metabolism. Those found to be of particular importance in wild-type JM109 were formate, succinate and acetate that are organic acids present in or at the end of the TCA cycle respiratory pathway. However, JM109-Thy^r^ had decreased levels of these metabolites and significantly increased lactate and pyruvate family amino acids. This is compelling evidence of a switch from respiration to fermentation as part of the strategy of *E. coli* to survive assault with polyphenols. The conclusion here is that increased tolerance to thymol is associated with a shift away from respiration to fermentation or the inability to enter the TCA cycle in JM109-Thy^r^ strain which may explain why it grew slower than the wild-type even without thymol. Our finding is similar to a study that used vanillin, which is a phenylpropene phenolic aldehyde, where the mechanism of vanillin antibacterial action was associated with inhibition of respiration in *E. coli* whilst in some lactic acid bacteria it disrupted K^+^ and pH homeostasis [26]. Moreover, a reported analysis of the metabolome of *E. coli* 555 by ^1^H NMR spectroscopy at different concentrations of carvacrol showed that although adaptation to carvacrol at sub-lethal doses was different from that occurring at higher doses, towards the higher concentrations of carvacrol there was a shift from respiration to fermentation [27]. Together these findings and those from our study suggest that *E. coli* exposure to phenolic compounds reduces growth which is accompanied by a shift from respiration to fermentation. It should be noted that lactate was already present in all samples tested suggesting some fermentation, possibly through hypoxia that occurred during growth or between harvesting and extraction. Moreover, there was little evidence of small metabolite leakage suggesting that at the concentration of thymol used (a modest 50μg l^− 1^) cell membrane damage was possibly minimal. Whilst this is not a direct evidence for the mechanism of action, it is an interesting possibility that phenolic compounds integrate in the cell membrane to disrupt electron transfer that is essential for respiration.

Genome sequencing analysis of JM109-Thy^r^ pointed towards two mutations leading to a potential loss of function of genes. Firstly, a non-sense mutation in the *acrR* gene encoding a repressor of the AcrAB efflux pump, and secondly a non-synonymous missense variant in the *rng* gene encoding ribonuclease G (RNase G). The acriflavine resistance regulator (AcrR) is a local transcription factor that regulates the expression of the outer and cytoplasmic membrane bound AcrAB-TolC multidrug efflux pump. The AcrAB-TolC multidrug efflux pump is involved in exporting a wide range of toxic compounds such as antibiotics, disinfectants, organic solvents and phytochemicals [28–31]. AcrR modulates the expression of *acrRAB* genes [32] and the associated AcrAB-TolC multidrug efflux pump [33]. The *acrR* gene is divergently located 141 bp upstream of the *acrAB* operon [32] and encodes a 215 amino acid long transcriptional repressor of the TetR family. The N-terminal domain of AcrR contains a DNA-binding motif, and the C-terminal domain has a unique sequence that is predicted to bind ligands [34]. The binding of drugs to the C-terminal domain of AcrR triggers a conformational change in the N-terminal DNA-binding domain resulting in the release of AcrR from DNA and allowing its transcription from its cognate promoter [35]. AcrR has long been implicated in organic solvent and antibiotic resistance in *E. coli* [36–42]. However, our reported mutation is novel and has not been reported before. In our JM109-Thy^r^ (Δ*acrR*) intracellular thymol accumulation was probably lowered by enhanced action of the AcrAB-TolC efflux pump due to loss of AcrR control. Loss of AcrR has been shown to result in the increased production of AcrAB-TolC efflux pumps and therefore, persistent clearance of thymol as highlighted by sustained growth of JM109-Thy^r^ in higher concentrations of thymol [36, 42]. In fact, Yuan et al., reported transcriptomic data supporting our findings in their thymol adapted *E. coli* O157:H7 bacterial model [8]. They found that thymol adapted *E. coli* O157:H7 had a significantly different transcriptomic profile under thymol stress with 113 downregulated genes limited to virulence, motility and replication genes, and 225 upregulated genes that included efflux pumps, stress response and iron transport genes. However, the limitation of that study is the absence of genome analysis to corroborate the altered expression genes are not harboring any mutations induced by thymol tolerance. Moreover, the limitation in our investigation is the lack of expression data in our evolved JM109-Thy^r^. In summary, inactivation of *acrR* is effective in increasing the MICs of thymol in *E. coli*. These results indicate that the AcrAB efflux pump plays an important role in survival against thymol. Most probably this mechanism in the comparative ‘resistance’ to thymol is the same mechanism created in response to the presence of antibiotics. Therefore, AcrAB efflux pump inactivation is a primary candidate for increasing bacterial sensitivity to antibiotics/ phytochemicals. It would be interesting to test this hypothesis by using specific efflux inhibitors such as phenylalanine arginyl *β*-naphthylamide (PA*β*N).

The other interesting mutation was in RNase G which functions in mRNA decay, tRNA and rRNA cleavage and maturation in conjunction with other RNase E and G family members [43]. *E. coli* RNase G was originally identified as an endoribonuclease involved in the maturation of 16S rRNA [44]. *E. coli* RNase G has been shown to be involved in the degradation of *adh*E mRNA encoding fermentative alcohol dehydrogenase [45, 46]. Different mutations reported in RNase G in the S1-like RNA-binding domain resulted in slowed growth of *E. coli* cultures [47]. Moreover, partial deletion of rng RNA-binding domain has been shown to enhance homoethanol fermentation [48]. It is possible that our reported missense mutation in RNase G that lies in the same domain would similarly support the metabolic shift to fermentation by alcohol dehydrogenase sustained expression and the noted slowed growth. Our study is limited by lack of a confirmation analysis of our reported genetic mutations causing thymol resistance in JM109, and the fact that our genetic findings are based on a single thymol resistant colony isolate. It is plausible that other colonies have adapted to thymol presence by other genetic and metabolic alterations. In addition, it is unclear if our reported mutations are contributing separately or in combination to thymol tolerance. An ideal confirmatory experiment would involve reintroduction of found genetic mutations in JM109 wild-type genetic background separately and in-combination to assess their individual and combined contribution to thymol resistance.

## Conclusions

Thymol resistance in *E. coli* is achieved by inducing morphological, metabolic and genetic changes. Despite the presence of ‘protective’ mutations against thymol the bacteria were very slow growing, were of low yield, and their metabolic profile suggests a shift to fermentation. It could be argued that when exposed to thymol *E. coli* would be rendered uncompetitive in the environments in which these bacteria are found which suggests that exposure to thymol will not readily select resistant tolerant derivatives in the ‘real world’. However, it is worthy to note that our observations are based on a single thymol resistant isolate, other isolates may have adapted by alternative mechanisms. If thymol and other EOs are used in complex environments they may pose little or even no threat of generating resistance unlike antibiotics. Whilst tempting to speculate EOs could be the new antibiotics of the future, much further work is needed.

## Methods

### *E. coli* adaptation to thymol test

*E. coli* K12 strain JM109 (New England BioLabs, Ipswich, MA, USA) was used for the thymol adaptation experiment. The test was performed after determining the minimal inhibitory concentration (MIC) [49]. Thymol was dissolved in ethanol 50% (v/v) to give a working stock solution of 5 mgl^− 1^. A primary thymol concentration of 100 μg l^− 1^ was used for the first exposure and thereafter increased by an additional 25 μg l^− 1^ increment so that the cells would be grown in a rising series of thymol concentrations (100–400 μg l^− 1^). For each cycle of growth 4.5 ml of each thymol concentration was added to Greiner CELLATAR® 96-wells plates. Five colonies of JM109 *E. coli* were taken from LB plates, inoculated into 10 ml of LB broth that was incubated aerobically shaking at 200 rpm at 37 °C for overnight. When growth was observed, 500 μl of the suspension adjusted to an OD600 = 0.02 (about 1 × 10^7^ CFU ml^− 1^) were added to each well for the first exposure in LB broth with 100 μg l^− 1^ thymol. The inoculated 96-well plate was incubated at 37 °C with shaking for 48 h after which a sample was streaked on to an LB agar plate and a 500 μl sample transferred to a fresh 96-well culture plate containing a concentration of thymol 25 μg l^− 1^ greater than in the previous well. This procedure was continued for 16 cycles at which time obvious growth was observed after 48 h of incubation at 37 °C. *E. coli* JM109 control cells for this experiment were grown in conditions similar to the above mentioned conditions throughout the 16 cycles without the addition of thymol. Both control and thymol treated cell were were plated on LB agar and colonies were picked and stored on cryobeads at -80 °C for subsequent experimentation.

### Growth rate assessment

The effect of thymol on the growth of trained tolerant and original *E. coli* JM109 was assessed by growing cells in 200 μl of different thymol concentrations in 96-well plate with 3 replicas, according to the CLSI M31-A3 guidance [50]. As a control, the last column of wells were inoculated without thymol as a negative control. The 96-well plate was covered with a lid and placed in an atmospheric control unit for microplate reader the FLUOstar Omega system (BMG LABTECH, Germany) at 37 °C with orbital shaking (200 rpm) and run for 24 h with spectrophotometric measurement (at 600 nm) every hour to determine bacterial growth. Immediately after 24 h incubation, 5 μl from each well was transferred to LB agar plates to determine the lowest concentration of thymol at which no growth could be observed after 24 h of incubation at 37 °C. This experiment was performed in triplicate with three repeats on separate days.

### Determination of bacterial morphology

JM109-Thy^r^ and original JM109 strains were observed by scanning electron microscopy (SEM). After overnight incubation in LB broth at 37 °C, bacterial cells were suspended to OD 600 = 0.5 in LB broth and divided into two sterile Eppendorf tubes to which thymol was added to one tube at a concentration of 100 μg l^− 1^, whilst the other was left untreated as a control. Samples were incubated in a rotary shaker set at 200 rpm and 37 °C. After 2 h, the cells were harvested by centrifugation at 14,000x g for 2 min, washed twice and suspended in phosphate buffer saline (PBS). Each suspension (200 μl) was placed on poly-L-lysine-coated glass cover slips for 15 min on both sides. Adhered bacteria were fixed with a solution of 2.5% glutaraldehyde pH 7 for 15 min. After fixation, samples were washed with water for 15 min, dehydrated by increasing serial dilution of ethanol (30, 50, 70, 80, 90%) immersions for 10 min each and for 1 h in 100%. Samples were dried in a Balzers critical point dryer CPD 030 (Bal-Tec, Germany), and metal coated in a sputter coater (Edwards, UK). All samples were observed with a field emission Quanta SEM equipped with a cold stage and a cryo-preparation chamber (Thermo Fisher Scientific, MA, USA). The experiment was performed in triplicate.

### DNA isolation and sequencing

Trained tolerant and original *E. coli* strain JM109 cultures grown for 18–24 h in LB were used for DNA extraction using yeast/bact kit (Qiagen, Germany) according to manufacturer’s protocol from fresh samples of bacterial cultures. The DNA concentration and quality were determined with a ND-1000 Nanodrop spectrophotometer (NanoDrop technologies, CA, USA). DNA stocks were adjusted to 100 ng/μl and stored at − 20 °C for sequencing. All centrifugation steps were performed at 14,000x g.

JM109 and derivatives were sequenced (Illumina, CA, USA) according to manufacturer’s protocols at 2 × 250-bp paired–end reads platform following Illumina library preparation. Raw sequence data were processed by an automated analysis pipeline, and reads were trimmed using Trimmomatic tool and the quality was assessed using in-house scripts combined with SAM tools, Bed Tools and BWA-mem. The genomes were assembled with SPAdes version 3.9.0 [51], and the assembly statistics were checked with Quast version 4.5 [52]. Comparison of the JM109 wild-type strain genome with JM109-Thy^r^ genomes was performed using Mauve multiple alignment program [53] and annotation with Prokka [54]. Results refer to positions on a reference *E. coli* genome as “universal” coordinates using the first published K-12 genome the *E. coli* MG1655 strain. MG1655 sequences were retrieved from GenBank (www.ncbi.nlm.nih.gov/nuccore/NC_000913.3) with accession number NC_000913. The *E. coli* MG1655 genome has been completely sequenced and the annotated sequence, biochemical information, and other available information were used to reconstruct the *E. coli* metabolic map [55].

### ^1^H nuclear magnetic resonance (NMR) spectroscopy

Prior to analysis, frozen stock suspensions of wild-type *E. coli* JM109 and JM109-Thy^r^ were cultured overnight in 5 mL of LB medium at 37 °C with shaking at 200 rpm. For the NMR metabolomics analysis, 200 μl of the overnight culture was re-inoculated in 10 mL of M9 defined minimal medium with glucose (0.2% w/v) as carbon source and thiamine supplement [56]. On the day of experiment, filtered M9 solution was supplemented with FeSO_4_ (2 μM/mL) and 1X trace metal mix solution (Sigma Aldrich, UK) and pre-warmed to 37 °C prior to inoculation as described. Subsequently the culture was incubated at 37 °C with shaking to an OD600 of 0.6 and was used for thymol treatment. Cultures were exposed to a sub-lethal concentration of thymol (50 μg l^− 1^), controls were cultured without thymol, and un-inoculated M9 media with or without thymol. There were 6 replicates for each of the treatments and incubation was for 24 h at 37 °C. Each 10 ml culture or control was centrifuged at 1000x g for 20 min at room temperature and 1 ml of supernatant samples were collected immediately afterwards and stored at − 80 °C until ^1^H NMR measurement. Supernatants were defrosted from − 80 °C and vortexed. A volume of 400 μl was transferred to a clean microfuge tube. Each sample was buffered with 200 μl phosphate buffer, vortexed and centrifuged at 14,000x g for 10 min, after which 550 μl of supernatant was transferred into 5 mm internal diameter NMR tube on the day of analysis.

^1^H NMR spectra were acquired on a Bruker (Bruker Avance III HD, UK) 700 MHz, using an automatic tuning-matching unit at 298 K, and an automatic sample changer. To facilitate compound identification, 1D spectra were acquired using standard Bruker 1D nuclear over Hauser enhancement spectroscopy (NOESY) pre-saturation pulse sequence on selected samples [57, 58]. After acquisition, spectra were manually phased, processed in order to realign spectrum phasing calibration on TSP at δ 0.00 ppm and baseline correction using MestReNova® software. Stacked spectra were imported into MATLAB (R2015b) MathWork® software where spectra were digitised between δ 0.5–10 ppm in order to delete useless information and avoid data bias; the region containing the water peak was deleted between δ 4.8 and 5.1. Peak assignment was done using online open access databases (chenomx® and HMDB) and 1D Spectra (for spectroscopy correlation) for molecule identification.

### Statistical analysis

For ^1^H NMR metabolic analysis, 6 samples were prepared respectively using 6 biological replicates. Multivariate statistical analysis was done using principal component analysis (PCA) plots to evaluate the metabolic variations existing between groups. Orthogonal projection to latent structure (OPLS) regression was performed on a minimum of 6 replicates per group, and between each group. PCA and OPLS correlation plots were produced to visualise differences in the metabolome between treatment groups. Loading and contribution plots were extracted to reveal the variables that bear class discriminating power. Moreover, to improve model visualization and interpretation, S-line plots were extracted to detect metabolites that influence variable selection as they display the overall importance of each variable (X) on all responses (Y) cumulatively over all components.

## Data Availability

The genome sequences generated and analysed during this study can be accessed after 1st of January, 2020; at (https://www.ncbi.nlm.nih.gov/genbank/) as BioProject PRJNA510551, with accession numbers RYWX01 (JM109 wildtype) and RYWY01 (JM109Rthy). Until then, the sequences are available from the corresponding author upon reasonable request.

## Abbreviations

acrR: Acriflavine resistance regulator
*E. coli*: *Escherichia coli*
EO: Essential oil
JM109-Thy^r^: JM109 thymol resistant derivative
MIC: Minimal inhibitory concentration
NMR: Nuclear magnetic resonance
OPLS: Orthogonal projection to latent structure
PaβN: Phenylalanine arginyl β-naphthylamide
PCA: Principal component analysis
SEM: Scanning electron microscope
TCA: Tricarboxylic acid
